# A deep sequencing reveals significant diversity among dominant variants and evolutionary dynamics of avian leukosis viruses in two infectious ecosystems

**DOI:** 10.1186/s12917-016-0902-6

**Published:** 2016-12-19

**Authors:** Fanfeng Meng, Xuan Dong, Tao Hu, Shuang Chang, Jianhua Fan, Peng Zhao, Zhizhong Cui

**Affiliations:** 1 College of Veterinary Medicine, Shandong Agricultural University, Taian, 271018 China; 2 Institute of Pathogen Biology, Taishan Medical College, Taian, Shandong China; 3 Poultry lnstitute, Chinese Academy of Agricultural Sciences, Yangzhou, Jiangsu China

**Keywords:** Subgroup J avian leukosis virus, Infectious ecosystem, 3-peptides LSD repeat insert (LSD^+^), Deep sequencing

## Abstract

**Background:**

As a typical retrovirus, the evolution of Avian leukosis virus subgroup J (ALV-J) in different infectious ecosystems is not characterized, what we know is there are a cloud of diverse variants, namely quasispecies with considerable genetic diversity. This study is to explore the selection of infectious ecosystems on dominant variants and their evolutionary dynamics of ALV-J between DF1 cells and specific-pathogen-free (SPF) chickens. High-throughput sequencing platforms provide an approach for detecting quasispecies diversity more fully.

**Results:**

An average of about 20,000 valid reads were obtained from two variable regions of *gp85* gene and *LTR-U3* region from each sample in different infectious ecosystems. The top 10 dominant variants among ALV-J from chicken plasmas, DF1 cells and liver tumor were completely different from each other. Also there was a difference of shannon entropy and global selection pressure values (ω) in different infectious ecosystems. In the plasmas of two chickens, a large portion of quasispecies contained a 3-peptides “LSD” repeat insertion that was only less than 0.01% in DF1 cell culture supernatants. In parallel studies, the *LTR-U3* region of ALV-J from the chicken plasmas demonstrated more variants with mutations in their transcription regulatory elements than those from DF1 cells.

**Conclusions:**

Our data taken together suggest that the molecular epidemiology based on isolated ALV-J in cell culture may not represent the true evolution of virus in chicken flocks in the field. The biological significance of the “LSD” insert and mutations in *LTR-U3* needs to be further studied.

**Electronic supplementary material:**

The online version of this article (doi:10.1186/s12917-016-0902-6) contains supplementary material, which is available to authorized users.

## Background

Avian leukosis virus (ALV) is an oncogenic retrovirus that induced lymphoid tumors in chickens and its genomic structure and molecular characteristics are well defined. It plays a critical role in the discoveries of reverse transcriptase, v-oncogenes and proto-oncogenes [1]. According to the host range, viral envelope interference and cross-neutralization patterns, avian leukosis viruses (ALVs) are classified into six subgroup (A to J) in chickens. ALV-J was first detected in meat-type chickens in the late 1980’s [2], and then spread globally [3–8]. So far, ALV-J is more pathogenic and mutate easily than other subgroups [9]. Although the eradication programs on ALV-J have been conducted in meat-type chickens since its discovery, it had spread into egg-type stock and the Chinese local breeds, which caused significant economic losses in China during the last 10 years [10–15].

Proteins gp85 and gp37 are encoded by the envelope gene of ALV, while gp85 protein constitute globular structures on the surface of the virus, which is closely associated with the process of viral binding and determine the specificity of subgroups. To understand molecular epidemiology of ALV-J among different types of chickens with various genetic backgrounds in many parts of the world, more than 200 ALV-J isolates have been subsequently sequenced and compared with gp85 region of envelope gene since late 1980s [4, 10, 16–23]. The early study suggested that gp85 sequences of the ALV-J strains isolated from different geographical areas and farms in different years showed highly variable and their similarity varied in the range of 80 – 100%. In terms of the *gp85* identity, the later isolates seemed to deviate gradually from the earliest isolate HPRS-103 [7]. However, many new isolates were obtained from different provinces of China after 1999. We found that no further deviate from HPRS-103 and all of their *gp85* sequences still varied in the same range [4]. There was also no evidence to show further sequence deviation from HPRS-103 even for the ALV-J strains isolated in the recent 10 years from layers or Chinese local breeds of chickens [10, 11, 13, 14, 24]. In addition, among 10 ALV-J isolates from ten individual layers with myelocytomas from the same flock demonstrated that they varied in the range of 80.3–97.1% in *gp85* region [25]. It seemed to suggest that there was no close relationship between ALV-J *gp85* homology levels and its pathogenicity or adaptation to different chicken breeds with different genetic backgrounds, although there were some epidemic phenomena indicated that ALV-J evolved to higher pathogenicity in different breeds of chickens.

In the past 30 years, almost all the molecular epidemiological data have been obtained by sequencing DNA fragments amplified and cloned from ALV-J infected CEF or DF1 cells. Such process would set up a bias for selection of certain quasispecies from the large population of viral particles in the given pathologic materials, for instance tumor tissues. By such selection, some significant variants associated with pathogenicity or adaptation to different genetic breeds may be survived by selective pressures. Wellehan reported that the dominant variants of San Miguel Sea Lion Virus populations altered significantly after its replication ecosystem switched from infected sea lions to cell cultures for 5 passages, the rare variants in sea lions became the dominant ones in cell cultures [26].

In this study, we analyzed and quantitatively compared dominant variants between ALV-J population replicated in infected chickens and cell cultures with the aid of deep sequencing-based method. The purpose of this study is to advance in understanding if cell culture ecosystem would cause selection pressures different from that of chickens with ALV-J infection, and whether the selection pressures would influence the evolution of ALV dominant variants. With these studies, we hope to identify specific epitopes or domains on *gp85*, or other genes, such as in the region of *LTR-U3*, which may associate with the differential selective pressures.

## Methods

### Sample preparation

The avian cell line DF-1 were obtained from the American Type Culture Collection (Manassas, VA, USA). These cells were grown in Dulbecco’s modified Eagle’s medium (DMEM; gibco, USA) supplemented with 10% fetal bovine serum and 100 mg/ml of penicillin and streptomycin. A liver with myloid tumors was collected aseptically from clinically hy-line variety brown with spontaneous infections and identified as ALV-J via virus isolation (Genbank: KR049171, KR049172). Tumor homogenate prepared in plasma-free DMEM was lysed, the supernatant was purified by high-speed centrifugation and 0.22-um-pore-size cellulose—acetate filtration. The resultant purified tumor suspension was designated as original liver suspension (Ori) which was used as the viral strain in laboratory experiments, and the concentration was about 1500 TCID_50_/100 ul.

In vitro group, 3000 TCID_50_ Ori were inoculated into DF-1 cells in logarithmic phase and maintained for 5 days as one passage. Then the infected DF1 cells were cultured via serial passages and cell free cultured supernatants were harvested at the 1st and 5th passage (P1 and P5). In vivo group, a total of 10 one-day-old specific pathogen free (SPF) chickens from the SPAFAS Co. (Jinan, China; a joint venture with Charles River Laboratory, Wilmington, MA, USA) were inoculated intraperitoneally with 3000 TCID_50_ Ori. The blood plasma collections were performed for virus isolation, while antibodies of ALV-J were detected at 2, 4 and 6 weeks post inoculation, respectively. Following inoculation, plasma was obtained from whole blood and stored at −80 °C. Two plasma samples free of antibody at 2 weeks of sampling and the cell culture from the 1st and 5th passages were chosen for high throughput sequencing (C1 and C2). The animal infection protocol was reviewed and approved by the Shandong Province Animal Ethics Committee.

### RNA extraction, RT-PCR and sequencing

Total viral RNA was extracted from samples of two ecosystems and original liver inoculum (Ori) using MagMAX Viral RNA isolation Kit (Life Technologies, USA) following the manufacturer’s instructions. Each sample was amplified using a forward primer with a six-digit error-correcting barcode as described earlier [27]. In addition, a 2-bp GT linker was added between the barcode and the 5′end of the forward primer to avoid a potential match between the barcode and the target sequences. Therefore, the forward primer was barcode-GT-primer, in which the barcode indicates the six barcode sequences that are specific to different samples, then three pairs of primers were designed according to the reference sequence HPRS-103 (Genbank: Z46390), namely gp85-A, gp85-B and LTR-U3 (Additional file 1: Table S1 and Figure S1). ALV-specific RT-PCR targeting the hypervariable region of the gp85 and LTR-U3 genes were then performed on the viral RNA using the two-Step RT-PCR Kit (TAKARA, China) at 42 °C for 45 min, 5 min denaturation at 95 °C, followed by 35 cycles of denaturation at 95 °C for 5 min, 95 °C for 30 s; annealing at 53 °C for 30 s, and extension at 72 °C for 30 s, with a final extension step at 72 °C for 10 min. The PCR products from both rounds were run of this reaction on a 1% agarose gels and scored. Bands of interest in the gels were cut out and the DNA was extracted from the gel using Qiagen Quick Gel Extraction Kit. The Products were quantified with a NanoDropND-1000 spectrophotometer (Thermo Fisher Scientific, Waltham, MA). A mixture of the amplicons was then used for sequencing on Illumina MiSeq platform according to the manufacturer’s instructions at the Beijing Genomics Institute (Shenzhen, China). A base-calling pipeline (Sequencing Control Software, SCS; Illumina) was used to process the raw fluorescent images and the call sequences, and data quality assessment were performed on the MiSeq instrument.

### Data analysis

Raw nucleotide sequences were filtered, aligned, trimmed and translated using pre-specified criteria applied uniformly. On average, there are 95% of the data above a quality value of Q30, which demonstrates a good quality of the demultiplexed reads. Then switch nucleic acid sequences of A, B fragment into amino acid sequences and threw away sequences with no biological significance for the following analysis (reads appeared more than 2 times were retained). The dominant *gp85* and *LTR-U3* variants of the samples were compared with each other under different infectious ecosystems using the Clustal W algorithm in MegAlign program of the DNASTAR package. Transcriptional regulatory elements in the U3 region were analyzed by the online service system of NSITE (Recognition of Regulatory motifs) of Soft Berry (http://www.softberry.com/berry.phtml). The statistical analysis was done by Duncan’s multiple range test.

In order to investigate quasispecies diversity under different ecosystems, we calculated the Shannon entrophy using clean reads of each sample.

Formula followed:

$$ {H}_{shannon}=-{\displaystyle {\sum}_{i=1}^{S_{obs}}\frac{n_i}{N} \ln \frac{n_i}{N}} $$

$$ \operatorname{var}\left({H}_{shannon}\right)=\frac{{\displaystyle {\sum}_{i=1}^{S_{obs}}\frac{n_i}{N}{\left( \ln \frac{n_i}{N}\right)}^2}-{H^2}_{shannon}}{N}+\frac{S_{obs}-1}{2{N}^2} $$

*S*_*obs*_ = The amount of haplotype observed by sequencing

*n*_*i*_ = Number of sequences for haplotype *i*

*N* = The total valuable sequence number obtained by sequencing

To minimize potential sampling bias and reduce the computation load, we performed a bootstrapping strategy for the clean reads of each sample. For each re-sampling with replacement, phylogenetic analysis was performed using RaxML [28] with 200 bootstrap replicates, under the GAMMACAT substitution model. All other parameters were set to their default values. Global selection pressure values (ω) were estimated using HyPhy method [29].

## Results

### MiSeq high throughput sequencing data

After several filtering steps, about 94 to 97% of the nucleic acid sequences (*LTR-U3*) or 86–93% of the amino acid sequences (*gp85*-A and *gp85*-B) from any sample of the raw reads were retained for subsequent analyses. The raw reads and the filtered reads obtained using MiSeq High-throughput Sequencing of the extracted RNA generated a median of more than 20,000 reads per sample (Additional file 1: Table S2).

### Comparison of the ratios of haplotypes in different viral infectious ecosystems

The ratios of sequence haplotypes to total valid reads for *gp85*-A, *gp85*-B and *LTR-U3* fragments from both plasmas of two chickens (C1 and C2) and DF1 cell culture supernatants of two different passages (P1 and P5) were decreased significantly as compared to that of the original liver inoculum (Ori) (Additional file 1: Table S3). The results suggested there might be some selective pressures on quasispecies of both *gp85*-A (hr1 and vr2 regions), *gp85*-B (hr2 and vr3 regions) and *LTR-U3* fragments when ALV-J from the Ori replicated in chickens or in DF1 cell cultures. Some variants in Ori were decreased dramatically to undetectable levels when replication ecosystem changed.

### Evolutionary dynamics of *gp85*-B (hr2 and vr3) under different infectious ecosystems

**Fig. 1:**
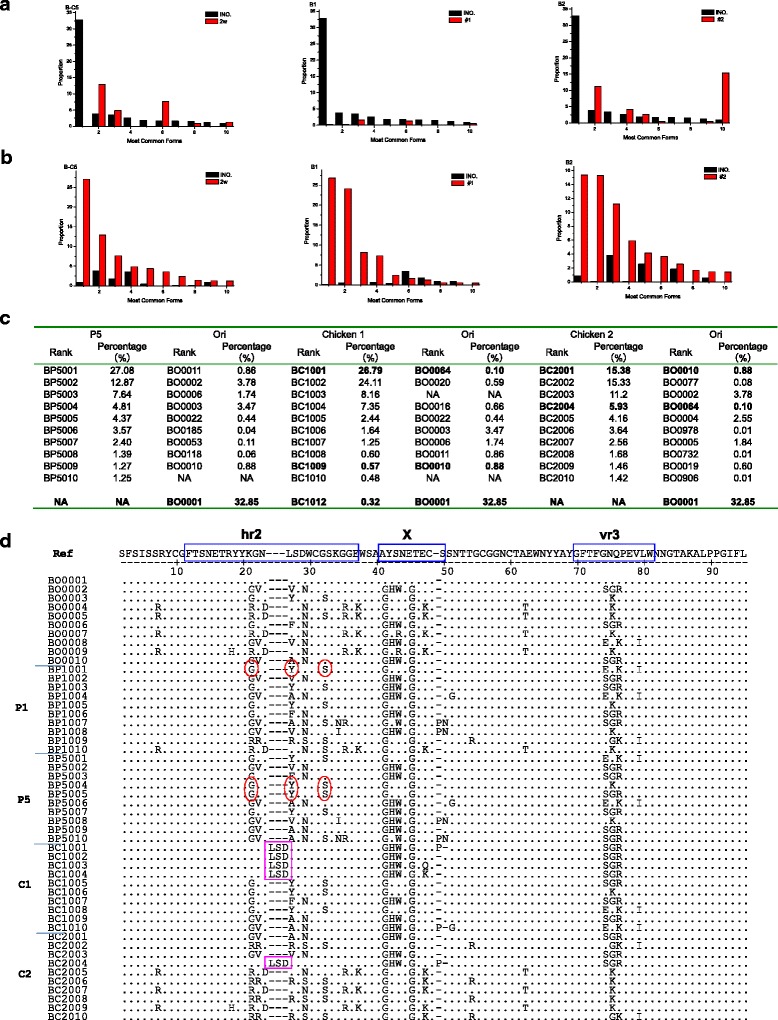
Evolutionary dynamics of *gp85*-B dominant quasispecies of ALV-J under different infectious ecosystems. **a** Percentages of the first 10 dominant quasispecies of gp85-B in the ori and their equals in cell culture supernatants of 5th passage (B-C5). Ori = black bars; chickens #1 (B1) and #2 (B2) plasmas = red bars. X-axis represents the first 10 dominant quasispecies of gp85-B in the original inoculum ordered based their ranks. Y-axis represents their percentages from total valid reads in each samples collected under different replication ecosystem. **b** Percentages of the first 10 dominant quasispecies of gp85-B in cell culture supernatants of 5th passage (B-C5) and their equals in the Ori. chickens #1 (B1) and #2 (B2) plasmas = red bars; Ori = black bars. **c** Haplotypes’ rank and their percentages of the first 10 dominant quasispecies of gp85-B in cell culture supernatants of 5th passage, plasma samples, and in the Ori. P5 = the cell culture supernatant samples of 5th passages; chicken 1 = plasma of #1 infected chicken; chicken 2 = plasma of #2 infected chicken, Ori = original liver inoculum. **d** The gp85-B amino acid alignment of the first 10 dominant quasispecies for viral samples collected in different replication ecosystyms. The dots indicate identical residues; the letter indicate amino acid substitutions; the dashes indicate gaps produced in the alignment; the blue square indicates two known variable regions (vr3 and hr2) and a new possible variable region X. The most dominant quasispecies (BO0001,32.85%) in the original inoculum (Ori) is used as the reference sequence on the top line corresponding to the amino acids sites #163-264 of *gp85* in ALV-J prototype HPRS-103 [Genbank:Z46390]

The dominant variants of gp85-B altered dramatically after replication under the two different ecosystems. The percentage of the most dominant variant of *gp85*-B in Ori decreased to a very low level and even became undetectable in infected chickens or cell culture supernatants, while some other sub-dominant variants were increased and decreased at a high and low percentages (Fig. 1a). Actually, the top 5 dominant variants in chicken plasmas or cell cultures were rare ones in Ori (Fig. 1b). It suggested that there were some strong selective pressures having influence on the evolution of dominant variants of *gp85*-B from infectious ecosystems.

The most dominant variant (BO0001) accounting for 32.85% in the Ori did not appear among the first 10 dominant quasipecies in either cell culture supernatants or chicken plasmas. We also find that there are two identical variants within the first 10 dominant variants of *gp85*-B from both chicken samples (C1 and C2). Sequences of the first dominant variant (BC1001) from C1 and the fourth dominant variant (BC2004) from C2 are 100% identical to the 64^th^ variant (BO0064) in the Ori. Similarly, the most dominant variant (BC2001) from C2 and the 9^th^ dominant variant (BC1009) from C1 are 100% identical to the 10^th^ dominant variant (BO0010) in the Ori (Fig. 1c). Although the other 8 dominant variants are different between the two chicken samples, but they have high homologies and only limited number of different sites than those from cell culture supernatants (Fig. 1d).

### Characterization of a specific domain of *gp85*-B associated with selective evolution of dominant variants under different viral replication conditions

The differences of amino acids in *gp85*-B dominant variants under different viral replication conditions are mainly in two known variable regions hr2 (aa#11–36) and vr3 (aa#69–80), or a new possible variable region X (aa#40–48). The most prominent difference is that the major dominant variants from chicken plasmas have a 3-peptides LSD repeat insert (LSD^+^) when compared to samples from the Ori or cell culture supernatants. Although the evolutionary dynamics of LSD^+^ in two chickens are not the same. In the plasma of chicken #1 (C1), all the top 4 dominant variants (BC1001, BCC1002, BC1003, BC1004) accounting for 66.32% from total valid reads get LSD^+^ at 2w post infection which is significantly increased compared to these equals in Ori consisting of only 1.35%. In another chicken (C2), only one as the 4^th^ dominant variant (BC1004) with LSD^+^ appears in the top 10 dominant variants, which accounting for 5.93% from the total valid reads, that was still a significant increase as the identical sequence haplotype (BO0064) in Ori was only 0.10%. In contrast, there is no LSD^+^ in the top 10 dominant variants in cell culture, and only 4 haplotypes with LSD^+^ consisted of only 0.02% in its total 29,986 valid reads are found among 3846 variants haplotypes, which was dramatically declined from 1.35% when compared to that only 163 haplotypes from 4047 variants haplotypes in Ori.

**Fig. 2:**
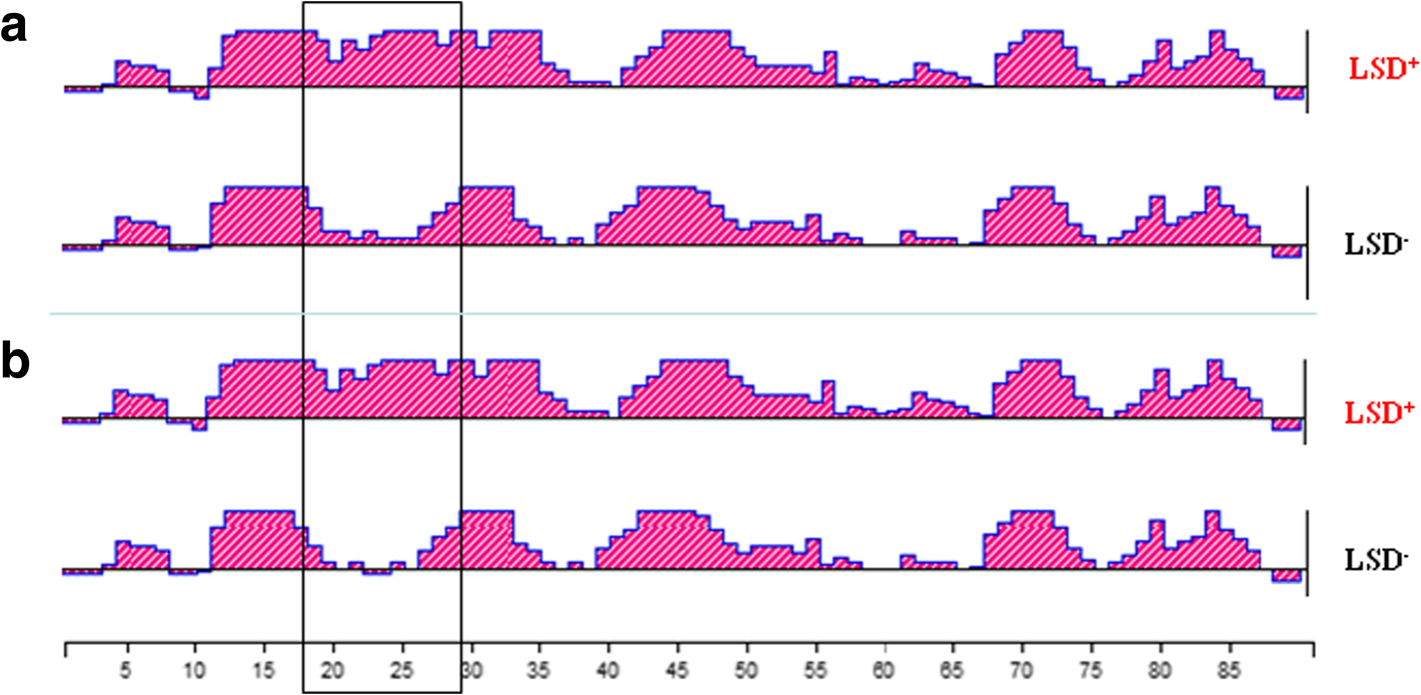
Comparison of the antigen index between the *gp85*-B quasispecies with or without LSD^+^. **a** The comparison of antigen index between 102001 (LSD^+^) and B102007 (LSD^−^). **b** The comparison of antigen index between B202004 (LSD^+^) and B202003 (LSD^−^). Antigenic profiles calculated with Jameson and Wolf (Jameson and Wolf, 1988) algorithm from the linear amino acid sequences, the different area of antigen index with or without LSD insertion mutation was marked with a black frame

All variants with LSD^+^ in different ecosystems were compared and analyzed. In C1, the top 10 LSD^+^ dominant variants accounting for 66.95% of the total valid reads, compared to only 1.45% in the Ori. While in C2, the first 10 dominant variants with LSD^+^ consisted of 6.62% of the total valid reads, but only 0.70% in the Ori. It indicates that variants with LSD^+^ were dramatically increased by positive selection after replication in chickens. Specifically, there are three completely identical variants with LSD^+^ in two chicken plasma samples (BC1001 vs BC2004, BC1002 vs BC2022, and BC1022 vs BC2040). However, evolution of variants with LSD^+^ are to the opposite direction after replication in DF1 cell cultures, that is, percentages of LSD^+^ positive variants decline rapidly and even disappear. Some LSD^+^ positive variants detected in infected chicken plasma are not detectable in the Ori even all reads are analyzed (Additional file 1: Table S4). There are some amino acid alterations in x and vr3 regions, but such variations trend to be convergent (Additional file 1: Figure S2). Also two pairs of LSD^+^ positive and LSD^+^ negative variants are compared for their antigenic index by computational analysis. The results indicated that LSD^+^ significantly increased the antigenic index in the new domain around the LSD insert (Fig. 2).

### Mutational analysis of *gp85-B* under different infectious ecosystems

**Fig. 3:**
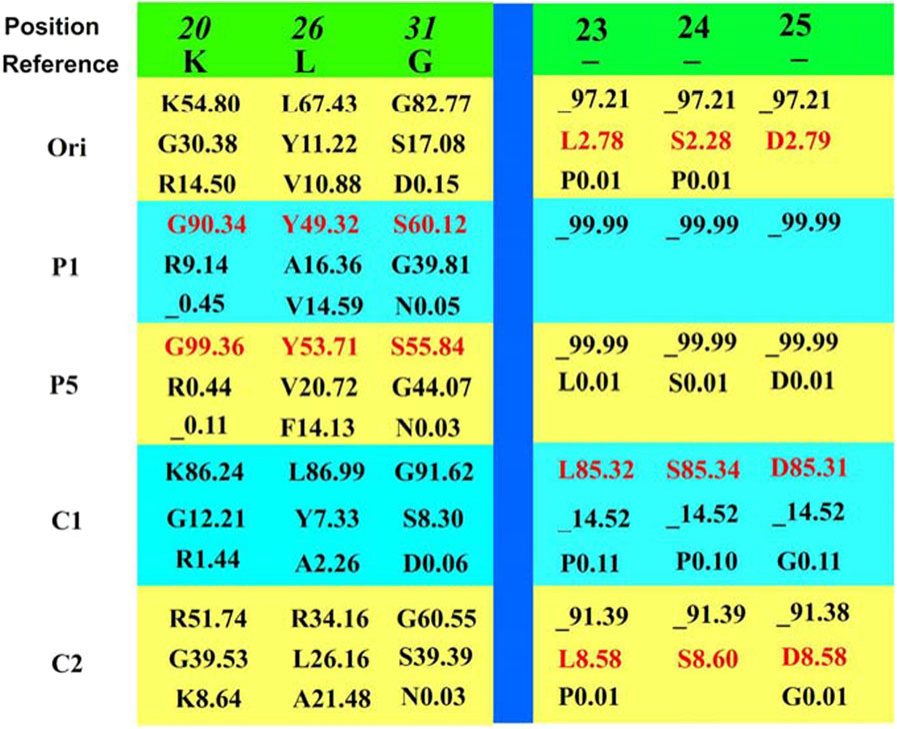
Comparison of amino acid changes in six sites on *gp85*-B under different infectious ecosystems. The first 3 frequent amino acids at each site and their percentages were listed for samples collected from the original inoculum. P1 = the 1^st^ passage cell culture; P5 = the 5^th^ passage cell culture; #1 = chicken 1; #2 = chicken 2. The capital letters indicate specific amino acid. The numerical numbers indicate the sites of the gp85-B by use of the most dominant quasispecies in the original inoculum as the reference

By analyzing the data from deep sequencing, mutational frequency of each site in the LSD^+^ domain is also compared independently among *gp85*-B quasispecies of ALV-J replicated in different ecosystems (Fig. 3). Each amino acid of L, S and D at the insertion sites (aa#23–#25) appears at the frequency of 2.78–2.79% in Ori, but it dramatically decreases to 0–0.01% after ALV-J is passaged in DF1 cells. In contrast, their frequencies were increased to 85.31–85.34% and 8.58–8.60% respectively in C1 and C2 which were very close to the frequency of 85.31 and 8.58% of the entire “LSD”, suggesting that the positive effect in infected chickens and negative selection in cell cultures were mainly associated with the intact 3-peptides insertion of LSD.

Besides, specific mutations of another 3 important sites in *gp85*-B were recognized to be associated with positive selective pressures in DF1 cells. The proportions of variants consisted of the 3 none-successive amino acids G-Y-S at aa positions #20, #26 and #30 were significantly increased, to 90.34, 49.32 and 60.12% in P1 and 99.36, 53.71 and 55.84% in P5 from 30.18, 11.02 and 17.08% in Ori, respectively (Fig. 3). In contrast, the proportions were 12.21, 7.33 and 8.3% in C1 and 39.51, 0.18 and 39.39% in C2. Further analysis of sequence data in Fig. 1 indicated that the 1^st^, 4^th^, 5^th^and 7^th^ ones among the top 10 dominant variants in 5^th^ passage cell culture contained the none-successive G-Y-S and consisted 38.96% of total reads, while two of the first 10 dominant variants in the original liver inoculum contained the G-Y-S which accounting for only 5.21% of total valid reads.

### Comparisons of the Shannon entropy and global selection pressure values (ω) under different infectious ecosystems

To roughly quantify the pressures that quasispesis underwent under different infectious ecosystems, we calculated the Shannon entropy and the global selection pressure values (ω). Our results showed the Shannon entropy in Ori was the highest, but when inoculated into chickens or DF1 cells different degree of decline were observed (Table 1). On the global selection pressure values (ω), those in cells were relatively stable at about 0.61, bigger than that from the Ori, but there were two different situations in the chickens ecosystem. Specifically, the ω values of the quasispecies in C1 were higher than those in P1 and P5, but the Shannon entropy is lower. Moreover, the ω values of the quasispecies in C2 were lower than those in P1 and P5, however the Shannon entropy is higher.

**Table 1:** The Shannon entropy and global selection pressure values (ω) under different infectious ecosystems

Ecosystems	The Shannon entropy	Global selection pressure values (ω)
Ori	4.90	0.59 ± 0.03^A^
C1	3.65	0.63 ± 0.04^B^
C2	4.52	0.54 ± 0.04^C^
P1	4.40	0.61 ± 0.04^D^
P5	4.24	0.61 ± 0.04^D^

Each value are calculated by 200 bootstrap re-samples of the distribution of variants. Each column in the upper label with different letters mean significant difference on Duncan’s multiple range test (*P* < 0.01)

### Comparisons of dominant variants in *LTR-U3* region under different infectious ecosystems

**Fig. 4:**
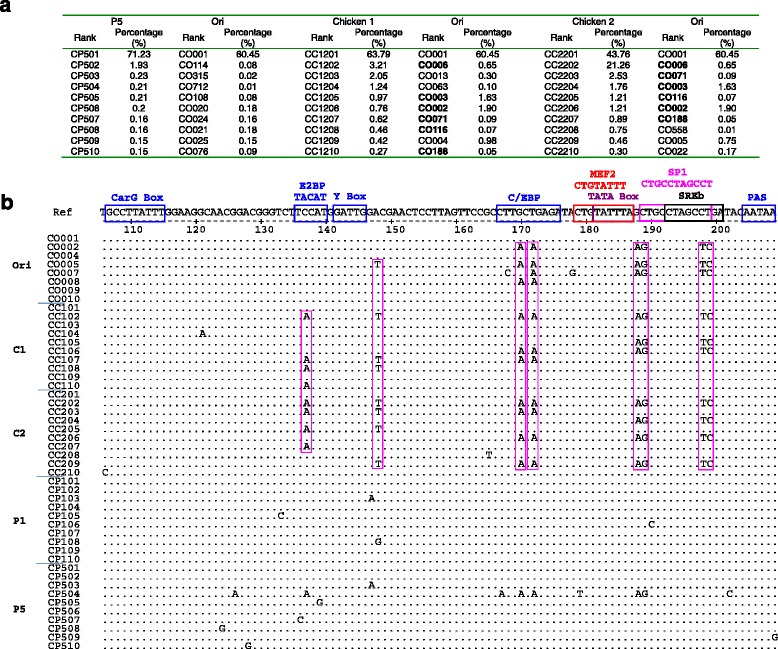
Evolutionary dynamics of the first 10 dominant quansispecies of *U3* fragment in different infectious ecosystems. **a** The first 10 dominant quasispecies haplotype (in the order according ranks) and their percentages of total valid reads in DF1 cell cultures (combined two passages, the left part). CC1001 = the quasispecies ranked the first in segment C from chicken 1; O0 = the original liver inoculum; C2 = chicken 2; P1 = passage1; P5 = passage 5 in cell culture. The last 3 numbers represents their ranks in the quasispecies population in each sample. **b** Base sequence alignment of the first 10 dominant quasispecies of U3 detected in each different infectious ecosystems. The reference sequence is the most dominant quasispecies in the original inoculum (CO0001, 60.45%), corresponding to bases #122–225 of LTR of ALV-J prototype strain HPRS-103. The dots indicate identical residues, while the letters indicate amino acid substitutions. The motifs as transcription regulatory elements were labeled on the top

The high throughput sequencing of *LTR-U3* region showed that the most dominant variants had not changed in both chicken plasmas and DF1 cell culture supernatants when compared to the Ori. However, the other sub-dominant variants of *U3* region evolved into different directions under different infectious ecosystems, in vivo and cell cultures. There were 7 of the top 10 variants were exactly the same in two chicken plasma samples which consisted of 70.08 and 72.62% of the total valid reads in the two chickens respectively (Fig. 4a). But all the left 9 major dominant variants of *U3* in cell cultures were completely different from those of chicken plasma.

Sequence alignment analysis demonstrated that *U3* region was more conservative in ALV-J replicated in cell cultures than in infected chickens, as the other 9 major sub-dominant variants haplotypes from DF1 cell culture supernatants had fewer mutations than that from 2 chicken plasma samples when compared to the most dominant variant common in different infectious ecosystems. More importantly, the most base alterations were located within some motifs as transcription regulatory elements (Fig. 4b) in samples from two chickens and the Ori. There were eight common mutation sites and similar replacement in the ecosystems of the two chickens, and then C/EBP, SP1, MEF2 and SREb regulatory elements losing their integrity but made one more motif of E2BP due to the replacement of C to A at the site #137. Obviously, it is due to differences of infectious ecosystems.

## Discussion

The object of this study was to understand if there is any influence from different infectious ecosystems on ALV-J dominant variants evolution. Both chickens and DF1 cell cultures were inoculated with the same Ori. The RT-PCR products of each sample were directly sequenced and analyzed by the deep sequencing, it produced extreme large sequence data covering genome variants even at very low frequencies. With the technology progresses on diversity in quasispecies and its evolutionary dynamics were obtained under different immunoselective pressures, antiviral drugs, and various viruses, such as HIV [30–33], and hepatitis B, C, and E viruses [34, 35] and some animal viruses [36].

Deep Sequencing generated a median of more than 20,000 reads per sample, which were large enough to compare and understand the quasispecies diversity from these samples. The ratios of haplotypes/valid reads of 3 fragments from chickens and cell culture samples were decreased compared to the Ori, suggesting that both infectious ecosystems demonstrated a negative selective effect on some quasispecies in the Ori. Among the 3 fragments sequenced, gp85-A fragment was less influenced by selective pressures, but gp85-B fragment was significantly influenced. Some dominant variants in the Ori was dramatically decreased in the inoculated chicken plasma and cell culture supernatant samples, but some very rare variants became the dominant ones. The results provided the direct experimental evidence that the infectious ecosystems would dramatically influenced the evolution of viral quasispecies. It is clear that the Ori was liver suspension and its ALV-J quasispecies mainly replicated in liver-associated cells, however, viruses in chicken plasmas or cell culture supernatant came from all kinds of sensitive cells in the body or replicated only in DF1 cells after the Ori was infected chickens or cell cultures. Bioinformatic analysis results showed the Shannon entropy in Ori was the highest, but when inoculated into chickens or DF1 cells different degree of decline were observed, which indicated there were some pressures in the ecosystems. On the global selection pressure values (ω), there was a big individual difference in chickens, but relatively stable in DF1 cells. There was significant difference (*P* < 0.01) between the two groups from chickens and DF1 cells, which also suggested different selective pressure in the two groups.

The envelope protein gp85 is related to recognition and adhesion to sensitive cells, and also is the major antigen for viral neutralization [9]. The diversity in *gp85 *sequence, especially epitopes at certain sites may influence the tropism of virus quasispecies to different types of cells in chicken body. For example, it has been reported that ALV-J prototype HPRS-103 has a low tropism for bursal follicles cells but does replicate well in cultured blood monocytes [9]. The most interesting result in this study is the discovery of the 3-peptides “LSD” repeat insertion (LSD^+^) in novel dominant variants of gp85-B fragments emerging in chicken plasmas samples, which increased the antigen index in the sub-region. However, there was no LSD^+^ positive variants among the top 10 dominant variants in two DF1 supernatant samples. Referencing to the principle of site-by-site positive selection analysis using the two rate fixed-effects likelihood (FEL) method [37], after artificial calculation we found that the LSD^+^ were under positive selection in chickens, while negative selection in DF1 cells. It might help to explain how evolution of different variants with specific epitope could be influenced by some selective pressures from ecosystem or its infectious ecosystems such as different organs, tissues or cell types. We speculate that variation in gp85 sequence similarity may not necessarily reflect its relationship to evolution in terms of higher pathogenicity to different genetic breeds of chickens, but some specific epitopes or domains on gp85 would influence.

*LTR-U3* region of ALV has only about 250 bp but contains several biological active motifs and enhancers influencing transcription and virus replication [38, 39], also it is a fragment easy to mutate on the ALV genome. However, analysis of deep sequencing data of *U3* region in different samples demonstrated that the viral population in chicken plasma samples came from ALV-J replicated in different types of cells, organs and tissues of the chicken and experienced quite different ecosystem selection pressures. Chicken plasma samples and DF1 supernatant sample had the same most dominant variants of *U3* as the Ori, which indicated that different infectious ecosystems did not have as high selective pressures on the evolution of *U3* quasispecies as that of *gp85*-B. But in the two chicken plasmas there were 6 absolutely identical variants for the top 10 sub-advantage variants while no reapeat with the top 10 sub-dominant variants in cell culture. Although the first 10 dominant *U3* variants were very conservative in cell culture supernatants, several sub-dominant variants from chicken plasma samples and Ori had mutations in its regulation elements C\EBP, SP1, MEF2 and SREb. Concerning the biological significance, its needs to be further investigated and studied.

## Conclusions

In conclusion, this study is the first to explore the replication of ALV-J in different ecosystem using deep sequencing technique. We found that significant differences in dominant variants and their evolution dynamics of gp85 from ALV-J in infected chickens or cell cultures. Especially, a tri-peptides “LSD” insert associated with positive selective pressures in infected chickens and negative selective pressures in DF1 cell cultures in *gp85* were identified. It suggests that the replication ecosystem has a significant influence on the evolution of viruses. The molecular epidemiology studies based on the isolated ALV-J in cell culture may not represent the true evolution of these viruses in infected chicken flocks in the field.

## Additional file

Additional file 1: Table S1. Three pairs of primers for Miseq High-throughput Sequencing. **Table S2** The numbers of raw reads and clean reads in each sample. **Table S3** Variations of ratios of sequence haplotype numbers to total valid reads in different replication ecosystems. **Table S4** Dynamics of the first 10 LSD+ positive quasispecies of gp85-B in different replication ecosystems. **Figure S1** The structure of env and LTR in ALV-J. **Figure S2** Amino acid alignment of top 10 LSD+ positive quasispecies of gp85-B in different replication ecosystems. (DOC 311 kb)

## Abbreviations

LSD^+^: A 3-peptides LSD repeat insert
ALV-J: Avian leukosis virus subgroup J
P1 and P4: The 1^st^ and 5^th^ passage
C1 and C2: Chicken 1 and chicken 2
Ori: Original liver suspension
SPF: Specific pathogen free
