# Supplementary material for: A deep sequencing reveals significant diversity among dominant variants and evolutionary dynamics of avian leukosis viruses in two infectious ecosystems
Source: BMC Vet Res. 2016 Dec 19;12:287. doi: 10.1186/s12917-016-0902-6 (PMC5168851; doi:10.1186/s12917-016-0902-6)
Supplement: Additional file 1: Table S1. — Three pairs of primers for Miseq High-throughput Sequencing. Table S2 The numbers of raw reads and clean reads in each sample. Table S3 Variations of ratios of sequence haplotype numbers to total valid reads in different replication ecosystems. Table S4 Dynamics of the first 10 LSD+ positive quasispecies of gp85-B in different replication ecosystems. Figure S1 The structure of env and LTR in ALV-J. Figure S2 Amino acid alignment of top 10 LSD+ positive quasispecies of gp85-B in different replication ecosystems. (DOC 311 kb) [file 12917_2016_902_MOESM1_ESM.doc]

**Table S1. Three pairs of primers for Miseq High-throughp**ut Sequencing.

| **Segments** | **Primer name** | **Primer sequence** | **Primer position corresponding to HPRS-103** | **Target gene** | **Target gene length(bp)** |
| --- | --- | --- | --- | --- | --- |
| A | *gp85*-A-F | GGCATTCCACAGTATCCTC | 5596-5614 | vr2 and hr1 | 327 |
|  | *gp85*-A-R | CGTCCATGATTGGTTGACA | 5940-5958 |  |  |
| B | *gp85*-B-F | GTCCAATAAACGTAGAGAG | 5966-5984 | vr3 and hr2 | 270 |
|  | *gp85*-B-R | GCCCTGTCCCCACAAATCA | 6251-6269 |  |  |
| C | *LTR*-C-F | GGGGAAATGTAGTGTTATGC | 7512-7531 | LTR-U3 | 250 |
|  | *LTR*-C-R | TGTGGTGGGAGGTAAAATGGCGT | 7741-7763 |  |  |

**Table S2.**  The numbers of raw reads and clean reads in each sample.

| Number | Segment A | | | Segment B | | | Segment C | | |
| --- | --- | --- | --- | --- | --- | --- | --- | --- | --- |
|  | Raw Data | Clean Data | Percentage | Raw Data | Clean Data | Percentage | Raw Data | Clean Data | Percentage |
| Ori | 27871 | 24154 | 86.66% | 22966 | 19803 | 86.23% | 14843 | 13963 | 94.07% |
| P1 | 20938 | 19107 | 91.26% | 18694 | 17012 | 91.00% | 6442 | 6160 | 95.62% |
| P5 | 49775 | 45865 | 92.14% | 32949 | 29986 | 91.01% | 30418 | 29360 | 96.52% |
| C1 | 34653 | 32030 | 92.43% | 26808 | 24986 | 93.20% | 27933 | 26824 | 96.03% |
| C2 | 46267 | 43089 | 93.13% | 15452 | 14311 | 92.62% | 27515 | 26285 | 95.53% |

C1 and C2: The plasmas of two infected chickens; P1 and P5: Cell culture supernatant samples of 1^st^ and 5^th^ passages; Ori: The original liver inoculum.

**Table S3. Variations of ratios of sequence haplotype numbers to total valid reads in different replication ecosystems**.

| Segments | Base or AA | Replication Ecosystems | | Haplotypes/total valid reads（%） | | Rangeability (%) |
| --- | --- | --- | --- | --- | --- | --- |
|  |  |  |  | Ori | Replication ecosystems |  |
| *gp85*-A | Amino  Acid | Chicken  Plasma | C1 | 4911/24154（20.33） | 3656/32030（11.41） | -8.92 |
|  |  |  | C2 |  | 5178/51398（10.07） | -10.26 |
|  |  | Cell  Culture | P1 |  | 2832/19107（14.82） | -5.51 |
|  |  |  | P5 |  | 453/45865（9.90） | -10.43 |
| *gp85*- B | Amino  Acid | Chicken  Plasma | C1 | 4047/19803（20.44） | 2576/24986（10.31） | -10.13 |
|  |  |  | C2 |  | 3250/21844（14.88） | -5.56 |
|  |  | Cell  Culture | P1 |  | 2798/17012（16.36） | -4.08 |
|  |  |  | P5 |  | 3846/29986（12.83） | -7.61 |
| *LTR*-U3 | Nucleic Acid | Chicken  Plasma | C1 | 1786/13963（12.79） | 2349/26824（8.76） | -4.03 |
|  |  |  | C2 |  | 2660/28500（9.33） | -3.46 |
|  |  | Cell  Culture | P1 |  | 750/6160（12.18） | -0.61 |
|  |  |  | P5 |  | 1998/29360（6.81） | -5.98 |

C1 and C2: the plasmas of two infected chickens; P1 and P5: Cell culture supernatant samples of 1^st^ and 5^th^ passages; Ori: The original liver inoculum. AA: The amino acid.

**Table S4. Dynamics of the first 10 LSD^+^ positive quasispecies of *gp85*-B in different replication ecosystems**.

| Cell | | Ori | | Chicken 1 | | Ori | | Chicken 2 | | Ori | |
| --- | --- | --- | --- | --- | --- | --- | --- | --- | --- | --- | --- |
| Rank | Percentage  (%) | Rank | Percentage  (%) | Rank | Percentage  (%) | Rank | Percentage  (%) | Rank | Percentage  (%) | Rank | Percentage  (%) |
| BP12316 | 0.01 | NA | NA | BC1001 | 26.70 | BO0064 | 0.10 | BC2004 | 5.93 | BO0064 | 0.10 |
| BP50517 | 0.01 | BO0020 | 0.59 | BC1002 | 24.11 | BO0020 | 0.59 | BC2022 | 0.27 | BO0020 | 0.59 |
| BP51741 | 0.00 | NA | NA | BC1003 | 8.16 | NA | NA | BC2039 | 0.10 | BO1174 | 0.00 |
| BP52173 | 0.00 | BO0016 | 0.66 | BC1004 | 7.35 | BO0016 | 0.66 | BC2040 | 0.10 | BO0723 | 0.01 |
|  |  |  |  | BC1022 | 0.15 | BO0723 | 0.01 | BC2042 | 0.08 | NA | NA |
|  |  |  |  | BC1023 | 0.14 | BO0161 | 0.05 | BC2155 | 0.03 | NA | NA |
|  |  |  |  | BC1025 | 0.13 | NA | NA | BC2198 | 0.03 | NA | NA |
|  |  |  |  | BC1027 | 0.12 | BP505 | 0.01 | BC2221 | 0.03 | NA | NA |
|  |  |  |  | BC1034 | 0.09 | BO3581 | 0.00 | BC2233 | 0.03 | NA | NA |
|  |  |  |  | BC1036 | 0.00 | NA | NA | BC2273 | 0.02 | NA | NA |

The last 3 or 4 numbers after represents their ranks in the quasispecies population in each sample. The LSD+ positive quansispecies haplotype names (in the order according ranks) and their percentages of total valid reads in DF1 cell cultures (combined two passages, the left part). C1 and C2: The plasmas of two infected chickens; P1 and P5: Cell culture supernatant samples of 1st and 5th passages; Ori: The original liver inoculum. chicken 1= plasma of #1;chicken 2= plasma of #2.

**
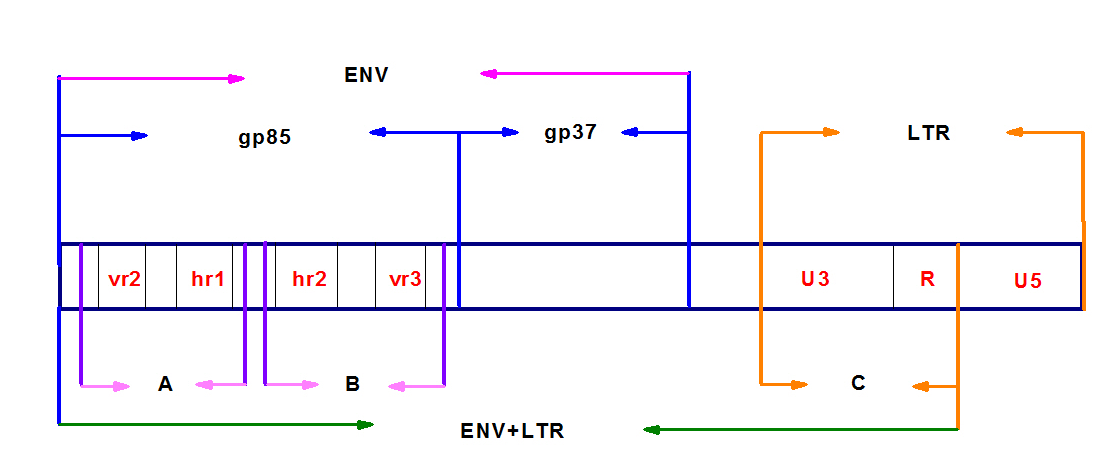
**

**Fig S1. The structure of env and LTR in ALV-J.**


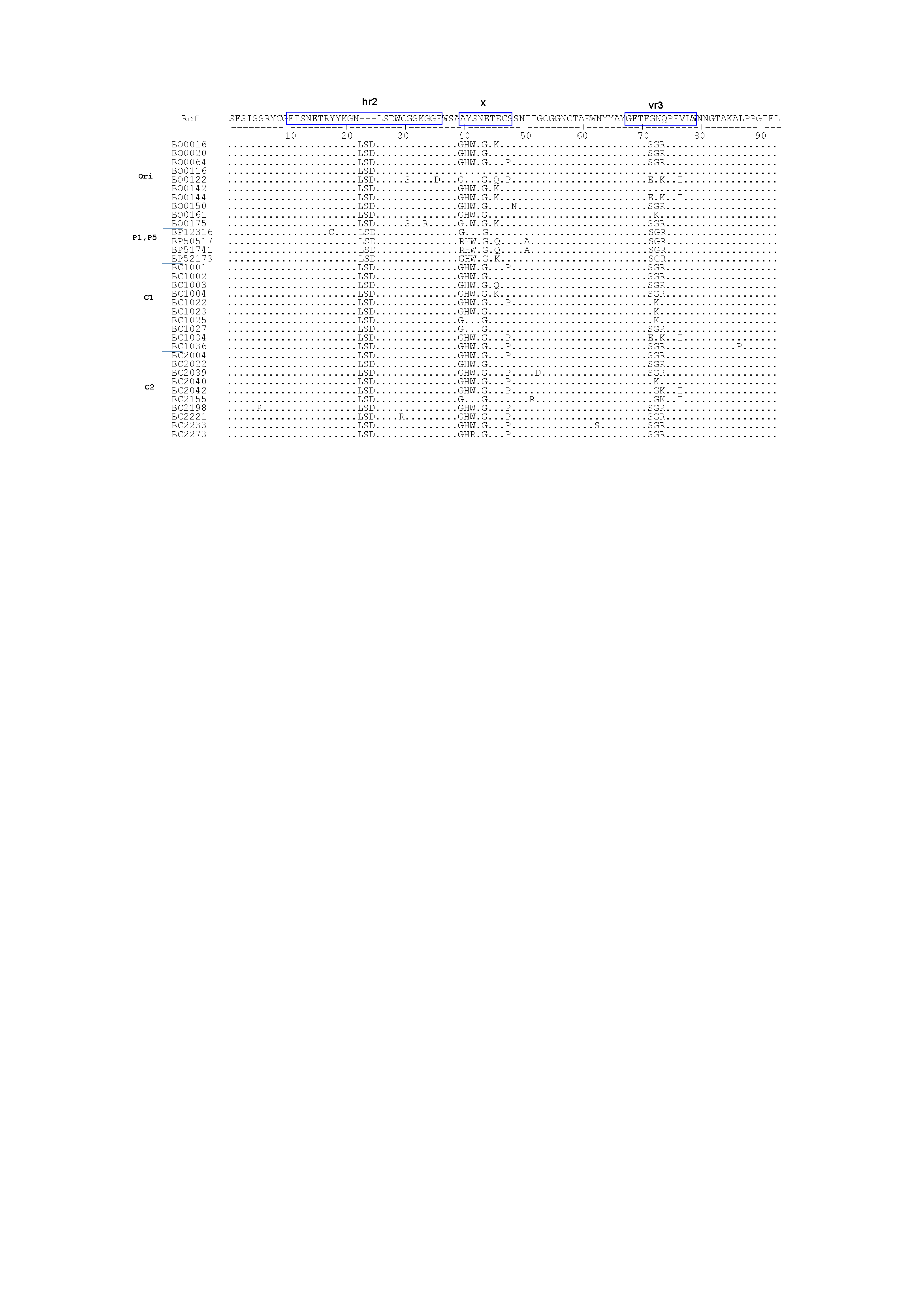


**Fig S2. Amino acid alignment of top 10 LSD^+^ positive quasispecies of gp85-B in different replication ecosystems.**

The last 3 numbers represents their ranks in the quasispecies population in each sample.The reference sequence is the most dominant quasispecies in the original inoculum (BO0001,32.85%) corresponding to the amino acids positon #163-#264 of gp85 in HPRS-103[Genbank:Z46390].The alignment of the sequences was done using the MegAlign program. The dots indicate identical residues, while the letters indicate amino acid substitutions. The dashes indicate gaps produced in the alignment. C1 and C2= the plasmas of two infected chickens; P1 and P5= cell culture supernatant samples of 1st and 5th passages; Ori= the original liver inoculum.
